# Effects of Climate Warming, North Atlantic Oscillation, and El Niño-Southern Oscillation on Thermal Conditions and Plankton Dynamics in Northern Hemispheric Lakes

**DOI:** 10.1100/tsw.2002.141

**Published:** 2002-03-08

**Authors:** Dieter Gerten, Rita Adrian

**Affiliations:** Leibniz-Institut für Gewässerökologie und Binnenfischerei, Müggelseedamm 301, D-12587 Berlin, Germany; Potsdam-Institut für Klimafolgenforschung, Telegrafenberg C4, D—14412 Potsdam, Germany

**Keywords:** climate change, freshwater ecosystems, North Atlantic Oscillation, El Niño-Southern Oscillation, lake ice, long-term, phytoplankton, zooplankton, large-scale synchronization

## Abstract

Impacts of climate warming on freshwater ecosystems have been documented recently for a variety of sites around the globe. Here we provide a review of studies that report long-term (multidecadal) effects of warming trends on thermal properties and plankton dynamics in northern hemispheric lakes. We show that higher lake temperatures, shorter periods with ice cover, and shorter stagnation periods were common trends for lakes across the hemisphere in response to the warmer conditions. Only for shallow dimictic lakes was it observed that deep-water temperatures decreased. Moreover, it became evident that phytoplankton dynamics and primary productivity altered in conjunction with changes in lake physics. Algal spring blooms developed early and were more pronounced in several European lakes after mild winters with short ice cover periods, and primary productivity increased in North American lakes. Effects of elevated temperatures on zooplankton communities were seen in an early development of various species and groups, as is documented for cladocerans, copepods, and rotifers in European lakes. Furthermore, thermophile species reached higher abundance in warmer years.

Obviously, the nature of responses is species specific, and depends on the detailed seasonal patterning of warming. Complex responses such as effects propagating across trophic levels are likely, indicating that observed climate—ecosystem relationships are not generally applicable. Nonetheless, the picture emerges that climate-driven changes in freshwater ecosystems may be synchronised to a certain extent among lakes even over great distances if climatic influences are not masked by anthropogenic impacts or differences in lake morphology. Macro-scale climatic fluctuations — such as the North Atlantic Oscillation or the El Niño-Southern Oscillation — were identified as the most important candidates responsible for such coherence, with the former predominating in Europe and the latter in North America. We emphasise, however, that the driving mechanisms and the future behaviour of these oscillations are rather uncertain, which complicates extrapolation of observed effects into the future. Thus, it is necessary to quantify the most important climate—ecosystem relationships in models of appropriate complexity. Such models will help elucidate the multiple pathways climate affects freshwater ecosystems, and will indicate possible adverse effects of a warmer future climate.

## INTRODUCTION

Weather and climate regulate physical, chemical, and biological processes in freshwater ecosystems at all time scales[1]. The physical features of lakes are most directly influenced by climate, as they are controlled by solar radiation, air temperature, and wind velocity[2]. Effects of ambient meteorological conditions on the biota of lakes are also well described. For example, the timing, composition, and intensity of the phytoplankton spring bloom in temperate lakes are tightly coupled to solar radiation, water temperature, and wind-induced turbulence[3,4]. Temperature, in particular, is of paramount importance for virtually all physiological and life-history parameters of aquatic organisms[2,5,6].

Over the long term, climate is considered the prime factor imparting variability to thermal conditions and biological processes in lakes, if pervasive human influences are absent[7]. In view of anticipated global warming (see Box 1), aquatic ecologists have made great effort during the past 10–15 years in exploring possible changes in freshwater ecosystems induced by recent warming trends or extreme weather conditions, or both. Current warming trends largely match projections of a warmer future climate (Box 1). Consequently, relationships established for recent decades are particularly valuable, because they indicate — at least qualitatively — the likely nature of ecological changes in the future[8]. Most of the studies were based on analyses of synchronous long-term records of ecological and limnological variables, respectively, which were derived from systematic ecosystem monitoring at a variety of sites particularly in North America and Europe. Some investigations were imbedded in international research programs, which had been founded to address explicitly the long-term dynamics of relevant variables in (aquatic) ecosystems (see, in particular, the Response of European Freshwater Lakes to Environmental and Climatic Change (REFLECT) project[9], the Long Term Ecological Research (LTER) programs[10], and the Freshwater Biological Association[11]).

Here we provide a review of observed decadal-scale effects of climate warming on lakes in the Northern Hemisphere. For the sake of brevity, we consider only impacts that were not fully covered by previous reviews, focussing primarily on impacts on thermal conditions and plankton dynamics. Excellent overviews already exist on the interaction of effects of climate warming with other anthropogenic impacts (e.g., activities in the catchments and their influences on lake chemistry, water pollution, overexploitation of resources[12,13,14,15,16,17,18,19,20], acid rain, and increased UV-B radiation[21]). We also do not discuss possible changes in biodiversity and biogeographical distributions of species, nor ecological invasions or species extinctions, which may happen in response to climatic change[22,23]. Climate warming effects on fish will not be considered here as well, since information on this topic has been published recently[12,24,25,26,27].

**BOX 1 — CLIMATE WARMING.** There seems little doubt that global climate warming is in progress, as the Earth's mean surface temperature increased by about 0.6 ± 0.2°C in the course of the 20th Century, including a maximum warming rate since around 1976[144,145]. The five warmest years in the Northern Hemisphere since at least 1400 A.D. all were recorded in the 1990s[146,147]. Much of this temperature increase is ascribed to the increasing long-term trends in anthropogenic emissions of greenhouse gases such as carbon dioxide and methane[145].

General Circulation Models project that the warming is likely to continue in the future. Global mean temperature is expected to increase by 1.4–5.8°C by the end of the current century, depending on the model and the underlying emission scenario[145]. The warming, however, will likely be accompanied by changes in local climate (temperature, precipitation, storm frequency, etc.), the magnitude and nature of which are still rather uncertain.

One of our objectives is to summarise effects of the two most dominant large-scale atmospheric circulation patterns controlling climate in large parts of the Northern Hemisphere: the North Atlantic Oscillation (NAO) and the El Niño-Southern Oscillation (ENSO) (Box 2). We aim to demonstrate that these circulation phenomena play an important role in synchronising changes among lake ecosystems over great distances, if climatic influences are not masked by anthropogenic impacts or differences in lake morphology.

**BOX 2 — NORTH ATLANTIC OSCILLATION AND EL NIÑO-SOUTHERN OSCILLATION.** A substantial fraction of the most recent warming in the Northern hemisphere was accounted for by a marked positive phase of a natural atmospheric circulation mode known as **North Atlantic Oscillation (NAO)**[145,148]. The NAO is a prime source of interannual and interdecadal climate variability in vast extratropical regions ranging from the eastern seaboard of the U.S. across the Atlantic up to Central Asia and north-western Africa[149,150,151]. Although the NAO is evident throughout the year, it is most pronounced during winter, explaining about one third of Northern Hemispheric temperature variability throughout December–March[122].

The NAO actually refers to a seesaw in air pressure differences between the subpolar Icelandic low and the subtropical Azores high, the two dominant centers of action over the North Atlantic[152,153]. Winters characterised by high air pressure gradients alternate at quasi-decadal intervals (6–10 years) with winters dominated by low gradients[154,155]. The NAO is commonly expressed in terms of an NAO index, which refers to the difference in sea level pressure anomalies between climate stations amidst the Azores high and the Icelandic low[116,156]; Hurrell's index is available via Internet[157]. High differences (indicated by a positive NAO index) usually produce a pronounced westerly wind component and predominating zonal circulation over the North Atlantic, yielding mild and rainy winters in West and North Europe, and cold winters in eastern North America. In contrast, low pressure gradients (negative index) cause a weaker and more meridional circulation pattern, likely resulting in cold winters over Europe, and mild conditions in parts of North America[154]. A high NAO phase dominated most of the winters during the most recent decade.

The rather simple NAO index can be easily applied to establish relationships between large-scale climatic variation and, for instance, ecological variables. The majority of NAO impact studies is based on Hurrell's index, which captures the pressure gradient between Stykkishólmur (Iceland) and Ponta Delgada (Azores)[116]. The NAO was identified recently as a main factor affecting ecological processes in both terrestrial and aquatic environments situated within the sphere of its influence[141].

The **El Niño-Southern Oscillation (ENSO)** is a recurring atmospheric–oceanic phenomenon taking place every few years across the tropical Pacific[158,159]. The most important manifestation of that oscillation are the warm ENSO events (El Niños), which are characterised by above-normal sea surface temperatures in the central and eastern equatorial Pacific during a period lasting for several months[160]. Measures of the ENSO are, for example, the NINO3 index[161] that refers to the sea surface temperature anomalies in the tropical Pacific, or the Southern Oscillation index[162], which is defined as the air pressure difference between Tahiti and Darwin (Australia).

ENSO is the most important driving mechanism of seasonal to interannual climate variability not only over tropical but also over vast extratropical regions, and the warming influence of El Niño on global temperature is now well attested[145,163]. Since the late 1970s, the warm ENSO state predominates, and the most dramatic El Niño events of the past century occurred in 1982 and 1983 and in 1997 and 1998[164,165]. These events were characterised, for example, by more zonal flow than normal and an eastward displacement of the Pacific Jet Stream, which resulted in very mild winters over much of northern and central North America, while precipitation patterns changed spatially inconsistently[107]. It should be borne in mind that the ENSO explains only a portion of winter temperature variability in North America[166,167], as regional phenomena and teleconnections with other circulation patterns such as the NAO exist[103,106,168]. ENSO influences are relatively weak over Europe[169,170].

Biological responses to El Niño episodes cover a wide range of ecosystems and taxa, including, for example, fisheries declines[171,172], range expansions of marine species[173,174], variations in discharge and associated changes in river ecology[175], or coral bleaching[164]. The signature of ecological ENSO impacts, however, is strongly regional[176].

## EFFECTS OF CLIMATE WARMING ON LAKE PHYSICS

The physical properties of water bodies are of utmost importance for most ecological processes in freshwater systems[2]. Ice formation, for instance, has a substantial influence on the underwater light, temperature, and turbulence regime[4,28]. Thus, changes in the duration of ice cover periods may induce significant changes in the inocula of phytoplankton, which in turn may affect the seasonal succession of plankton. Thermal stratification may also affect considerably the structure and composition of plankton assemblages. For example, in Rostherne Mere (U.K.), *Scenedesmus* predominated in the phytoplankton community in years when stratification was unusually stable while *Oscillatoria* predominated in years with frequent mixing[29]. The length of the stagnation period may furthermore affect nutrient availability. For small dimictic

Heiligensee (Berlin, Germany), it was found that more nutrients accumulated in the hypolimnion in years with early stratification onset[30]. Here we provide an overview of long-term changes in lake physics (water temperature, stratification behaviour, ice conditions) that were observed recently for lakes around the Northern Hemisphere.

## Effects on Thermal Properties

A number of climate impact studies demonstrated pronounced effects of recent warming tendencies on thermal habitats of freshwater ecosystems. Water temperatures usually increased in parallel with higher air temperatures. The first report of this phenomenon[14] documented that mean water temperatures of boreal lakes in northwestern Ontario increased by ≈ 2°C during the period 1969–1988. Subsequent studies provided further evidence that water temperatures of numerous lakes in the Northern Hemisphere increased significantly during the most recent decades (namely in winter/spring[30,31,32], summer[33,34,35,36], and autumn[37]).

Hypolimnetic water temperatures of thermally stratified lakes responded less consistently to climate warming trends, mainly because climate effects were modulated decisively by lake depth and morphology. Deep-water temperatures increased little or not at all at higher air temperatures[32,34,38]. Yet, accumulative multiannual warming is likely to occur in very deep, oligomictic lakes that do not fully circulate each winter[39,40]. The hypolimnia of shallow lakes tended to become colder in years with warm weather, probably as a consequence of earlier stratification at low lake temperatures[32].

The advanced onset of thermal stratification in lakes in response to rising air temperatures is documented by several studies[30,33,34]. There are also indications that the mixis regime of lakes was altered in warm years. Polymictic lakes stratified more permanently[41], and dimictic lakes were predicted to become more monomictic due to the lack of inverse stratification in ice-free winters[42,43].

Additionally, it was observed that metalimnia became sharper in warm years[33]. Moreover, mixing depths increased in small but not in large lakes in warm, bright summers. The main reason for this discrepancy is that mixing depth in large lakes (> ≈ 5 $km^2$) is controlled primarily by aspects of wind exposure, while in small water bodies, water clarity, cloud cover, and solar penetration are important[34,44,45]. Water clarity, in turn, will decrease, if fish and phytoplankton abundance[46] or DOC levels are high[47].

## Effects on Ice Conditions

Historical (decadal to centennial) records of ice freeze- and break-up dates are available for a variety of sites in temperate latitudes of the Northern hemisphere[48]. Analysis of these time series provides valuable information about lake responses to short-term and, particularly, long-term variations in the winter climate. A variety of studies documented that ice cover periods of lakes have shortened remarkably during the past decades in Wisconsin[49,50,51], western Ontario[14], New Hampshire[52], Siberia[53], Japan[54], Finland[55], Switzerland[56], Germany[57], and Sweden[58].

Specifically, significant changes toward earlier ice break-up dates happened both around 1890 and around 1980 at Lake Mendota (Wisconsin)[49]. For Müggelsee (Berlin, Germany), a 1°C increase in winter air temperature resulted in a reduction of ice coverage by about 17 days[59]. This reduction in ice coverage is similar to that recorded for North American lakes (Lake Mendota, 12.7 days; Grand Traverse Bay, Lake Michigan, 15.5 days [50]). Yet ice thickness, lake size, and latitudinal position modify these figures[60]. In addition to earlier break-up dates, later freeze-up dates were observed for Lake Suwa (Japan)[54] and for Mirror Lake (New Hampshire)[52]. Furthermore, the ice-free season is expected to extend dramatically in Arctic lakes such as Toolik Lake (Alaska)[61] while in lower latitudes, ice formation does not occur at all in extremely mild winters[43,50,59]. Overall, ice duration on lakes and rivers decreased consistently across the Northern Hemisphere during the past 150 years[62].

## EFFECTS OF CLIMATE WARMING ON LIMNETIC PLANKTON COMMUNITIES

Climatic conditions cause interannual and interdecadal variability in freshwater plankton communities[7]. Although physical features, anthropogenic impacts, and nutrient availability may be more important drivers for phytoplankton growth and abundance than direct climatic influences[60,63], climate exerts numerous indirect influences on aquatic food webs. Thus, global climate warming is anticipated to induce a number of interrelated effects across the trophic cascade in lakes around the globe[12]. Generally, it is expected that productivity may increase at all food web levels. In other words, effects of climate warming (e.g., increases in primary production, zooplankton biomass, and fish yields) will superficially resemble eutrophication[64]. In the following section, we discuss recent studies that document climate-related changes in freshwater plankton communities in the Northern Hemisphere.

### Effects on Phytoplankton and Primary Production

Climatically driven changes in phytoplankton abundance first became obvious in European freshwaters after a series of very mild winters in the late 1980s and early 1990s. Besides higher chlorophyll *a* concentrations during the winter season in small Heiligensee, the development of the spring bloom started about 1 month earlier in that lake. This was probably a consequence of shorter ice covers and, thus, better light conditions[30]. Additionally, the algal composition shifted toward a dominance of cyanophytes, as earlier stratification and earlier nutrient limitation prevented diatoms from becoming dominant[30,65].

In some other lakes, the phytoplankton composition in spring was dominated by diatoms after mild winters, and the spring bloom occurred early and was more pronounced (Plußsee (northern Germany)[66]; Müggelsee[57]; Lake Erken (southern Sweden)[58]). These changes also stemmed from the shortening of ice cover periods in mild winters, which favoured diatom growth owing to enhanced turbulent mixing and better light conditions. Furthermore, phytoplankton biomass in summer in Lake Erken was related to the timing of ice break-up[67]. The authors speculated that the earlier spring bloom after mild winters resulted in a longer mineralisation time, which in turn led to higher nutrient availability for the summer bloom.

Moreover, it became apparent that annual primary production in both Lake Tahoe (California) and Castle Lake (California) were related strongly to ambient weather conditions[68]. Primary productivity in the former was mainly determined by the maximal depth of spring mixing, which affected the quantity of nutrients returned from deep waters to the euphotic zone. On the contrary, annual productivity in Castle Lake was related to the amount of winter snowfall and spring precipitation. For example, productivity was low in years with much snowfall, which delayed ice break-up and the onset of the growing season. In addition, high amounts of subsequent spring rain led to the washout of substances involved in summer production. These processes affected particularly the hypolimnetic productivity in summer, whereas epilimnetic productivity in late summer was controlled by interactions on higher trophic levels[69]. Similarly, large Canadian Shield lakes had higher annual rates of primary production than small lakes, owing to the longer ice-free period and the deeper mixed layer of the former[70]. Long-term changes in primary productivity are, to our knowledge, not documented for lakes outside of North America, but the observed increases in vernal phytoplankton biomass in several European lakes indicates similar changes there[30,57,58,66].

## Effects on Zooplankton

Similarly to phytoplankton, zooplankton populations (rotifers and cladocerans) reached their spring maximum earlier after mild winters in some central European lakes[57,66,71,72]. The main reasons for these changes were either the climate-induced rise in algal availability, or warmer conditions in early spring, which led to early development of zooplankton. Magnitudes of zooplankton peaks, however, usually were independent of winter conditions[57,72], except for *Daphnia* in Lake Constance[73] (see below). Additionally, the dominance of colonial diatoms in Esthwaite Water (U.K.) in mild, windy winters resulted in high abundance of *Eudiaptomus gracilis,* while *D. hyalina* were more abundant in cold winters[74].

Furthermore, the clear-water phase — a typical drop in algal biomass in late spring in temperate meso- and eutrophic lakes — occurred about 2 weeks earlier in several lakes when water temperatures were above-normal during winter and spring (Plußsee[66], Lake Constance[71], Müggelsee[72]). Higher water temperature led to an earlier peak in *Daphnia* abundance, whose grazing pressure suppressed the preceding phytoplankton bloom. Even the timing of the decline of *Daphnia* abundance in July in Lake Constance was correlated with meteorological conditions in winter[71]. Contrarily, the occurrence and duration of the mid-summer decline of *D. galeata* in shallow polymictic Bautzen Reservoir (Germany) was related to warming events in spring and summer rather than in winter[75]. Specifically, the spring warming triggered an early and high abundance of daphnids, which led to high mortality rates due to aging and low reproduction during the resulting clear-water phase. The warming in early summer caused additional mortality by early growth and predation of planktivorous young-of-the-year fish.

Impacts of meteorological conditions in early summer on zooplankton communities were also described for Lake Windermere (U.K.), where zooplankton populations may be more effectively controlled by weather than by abundance of planktivorous fish[76]. In fact, biomass of crustacean zooplankton was high in summers following warm Junes. When the lake became stratified in June already, population of edible algae developed earlier than in other years. As a consequence, the algal population developed too early to match the zooplankton population, which thus became food limited. Interestingly, these processes were also correlated significantly with year-to-year variations in the northerliness of the Gulf Stream[77,78].

Higher spring biomass of *Daphnia* in response to climatic conditions was observed for Lake Constance as well. Other than in England, however, this phenomenon was coupled to high water temperatures in early spring, which led to a reproductive success of the zooplankton[73]. Furthermore, elevated temperatures in late spring and summer led to a prolonged active phase of several copepod species in Müggelsee. That is, the animals emerged early from diapause in spring and entered diapause somewhat later in autumn[79]. In fact, these temporal shifts in seasonal phenologies were induced primarily by the higher water temperatures in late spring while effects of mid-summer heat waves were minor[35]. Thus, other than parthenogenetically reproducing cladocerans, copepods with their rather complex and prolonged annual life cycle are likely to show time-lagged responses to warming events.

## Species-Specific Responses

Although many studies revealed climate effects at the level of functional or taxonomic plankton groups, it is most likely that the response of aquatic organisms varies at the species level. For instance, changes in the overwintering success of zooplankton in response to an autumnal warming trend differed among species[80]. Moreover, it was observed that zooplankton species in Heiligensee shifted from the large-bodied *D. galeata* to small *D. cucullata* in response to changes in the phytoplankton composition, which in turn had been induced by mild winters[65]. This observation is in agreement with that of Moore et al.[81], who found that high water temperatures caused reductions in body size of zooplankton populations. These shifts were mediated both directly and indirectly by other changes in the community, such as increased abundance of cyanobacteria at higher temperatures. In addition, abundance of *Cyclops vicinus* increased in Heiligensee due to the higher food availability in mild winters while *C. kolensis* decreased as a consequence of higher predation by *C. vicinus*[82].

Responses of cyclopoid copepods to warmer summer seasons also were clearly species-specific since only the most thermophile species exhibited higher peak abundance in those years, while *Acanthocyclops robustus*, a species adapted to a broad temperature range, was rather insensitive to the warming[35,79]. Moreover, the changes in the copepods' population dynamics depended on the detailed seasonal patterning of the warming, as they responded to warming events at certain times within the summer season only. For example, *Thermocyclops oithonoides* was influenced primarily by a warming in May, whereas *Mesocyclops leuckarti* was affected significantly by heat waves in mid-summer[35]. Laboratory experiments[81,83,84] suggest that secondary productivity of certain zooplankton species may have increased in response to climate warming, but long-term records to check that hypothesis are, to our knowledge, not available.

It is important to recognise that climate changes are likely to generate complex, and possibly time-lagged, direct and indirect responses in the entire food web[85,86,87,88], and these responses are often nonlinear [89]. Moreover, future environments may include novel combinations of (climatic) variables not encountered in the data used to analyse the present relationships. Extrapolations of the latter consequently must be done carefully[87]. Nonetheless, there is no doubt that temperature-sensitive aquatic organisms act as harbingers of climate warming[90]. This indicator function of single species in ecosystems undergoing climate change is well recognised in terrestrial ecology (e.g., breeding phenology of birds[91], tree phenology[92], insect abundance[93]). It is, however, much less well developed in plankton ecology (but see[65,71,82]).

Overall, long-term studies ultimately suggest that the typical seasonal succession in lakes[94] is currently advancing in many meso- and eutrophic lakes in temperate latitudes—namely, the seasonal course of water temperature, ice break-up and stratification onset, the phytoplankton spring bloom, the clear-water phase, and the phenology of sensitive plankton species. This phenomenon agrees with observations for terrestrial environments where species are responding to the recent warming trends by advancing the date of their growth, reproduction, or migration[91,95,96,97].

## LARGE-SCALE SYNCHRONISATIONS: THE ROLE OF THE NAO AND THE ENSO

Most climate impact studies cited herein report warming effects on single lakes. Yet, some studies provide strong evidence for a simultaneous response of multiple lakes at regional or even supraregional scales (see Fig. 1). These interlake synchronies usually are brought about by spatially coherent variation in climatic factors such as air temperature, precipitation, high-altitude cloud cover, and geostrophic wind velocity[31,36,98,99,100,101,102].

Therefore, aquatic ecologists should be alert to the possibility that changes in lake ecosystems may be synchronised by macroscale climatic fluctuations such as the NAO and the ENSO. In fact, the recent positive phase of the NAO contributed significantly to the elevated winter and spring surface water temperatures in lakes across Europe[31,32,71,74]. Moreover, the observed advancement in the ice break-up of lakes was controlled quasi-simultaneously by the NAO in a vast area covering, at least, southern Sweden[58], north-eastern Germany[72], Switzerland[103], and even Lake Baikal (Siberia)[53]. The NAO also affected plankton dynamics in several European lakes (Figs. 1, 2). The NAO-related early ice break-up (or the total lack of an

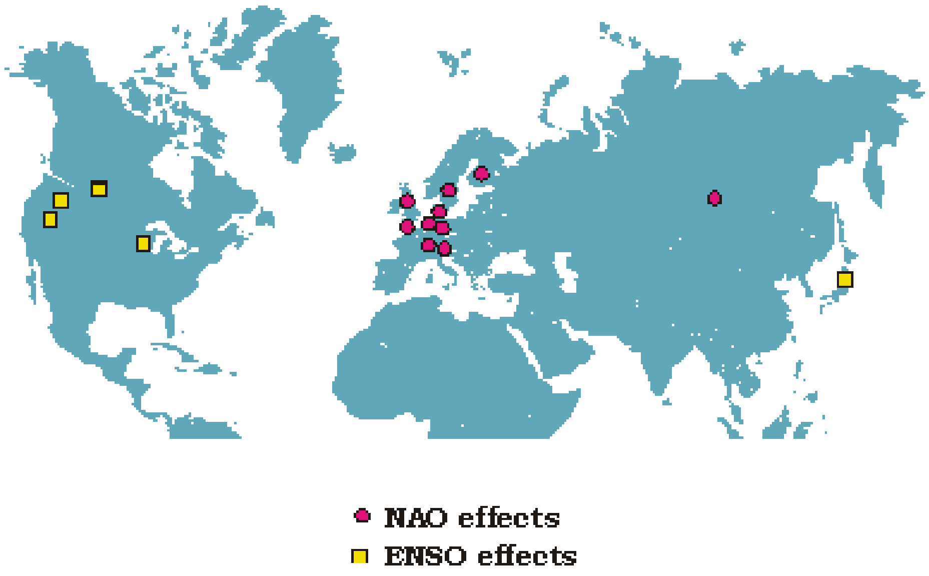

**FIGURE 1.** Locations of lakes in the Northern Hemisphere that were affected by either the NAO (red symbols) or the ENSO (yellow symbols). See text for references and processes involved.

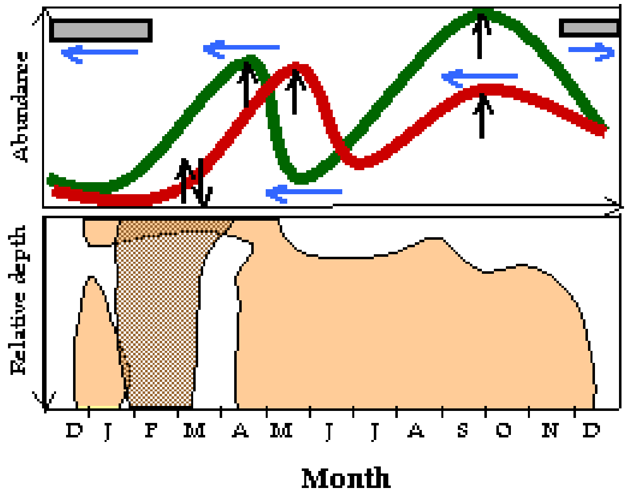

**FIGURE 2.** Schematic representation of warming impacts on freshwater ecosystems in temperate latitudes, based on long-term observations for a variety of lakes in Europe (see text for references). The **upper panel** illustrates effects on the seasonal plankton phenology in meso- and eutrophic lakes (green line, phytoplankton; red line, zooplankton; modified PEG-model[94]), and on the ice phenology (gray bar). Black arrows denote either increasing (↑) or decreasing (↓) abundance of plankton species or groups. Blue arrows indicate changes toward earlier (←) or later dates (→) of succession events. The **lower panel** indicates significant correlations ($p < 0.05$) between the winter NAO index and water temperature profiles of both a shallow polymictic (brown area) and a deep dimictic lake (orange area) (modified after Gerten and Adrian 2001[32]). Note that ENSO impacts on water temperature are likely to vary in a similar manner among lake types.

ice cover) in both Lake Erken and Müggelsee induced earlier and more pronounced algal spring blooms[57,58,59], which thus were related to the NAO as well[58,72]. It is likely that the enhanced phytoplankton biomass in Heiligensee[30] and Plußsee[66] after mild winters also was associated with the NAO.

For some lakes, NAO effects on plankton communities propagated into late spring. Specifically, the advancement in peak abundance of *Daphnia* and the timing of the clear-water phase in both Lake Constance[71,73] and Müggelsee[104] happened synchronously in response to the recent, positive NAO state, even though the lakes are located ≈ 700 km apart from each other. The suggested temperature-driven, large-scale coherence in the timing of the clear-water phase was supported by analysis of 71 Dutch and 21 central European lakes[105]. Even the timing of *Daphnia* population decline in summer in Lake Constance was related significantly to the NAO[71]. A further relationship between the NAO and zooplankton dynamics was reported from Esthwaite Water, where high NAO states accounted significantly for the overwintering success of *E. gracilis*[74]. Likewise, the above-described changes in population dynamics of single zooplankton species in Heiligensee after mild winters [55,82] probably were indirectly associated with the NAO.

El Niño events affected freshwater ecosystems in North America in several ways (Fig. 1). For instance, the extent of ice covers on the Laurentian Great Lakes is severely limited in mild winters following El Niño years[106]. As a consequence, the Great Lakes experienced one of the least extensive ice covers of the $20^{th}$ Century in the extreme El Niño years of 1997 and 1998 [107]. Overall, freeze-up and break-up dates from 62 lakes and rivers throughout the Northern Hemisphere (North America, Europe, Asia) were found to be related to the ENSO phenomenon[108].

The recent trends toward earlier break-up dates were spatially coherent in lakes across the Laurentian Shield, although coherence was highest for lakes with similar ice-off dates, and for lakes located at similar latitudes[109]. For example, the phenology of thawing was highly coherent among southern Wisconsin and south Dakotan lakes because these lakes integrated more or less the same seasonal weather. Ice-off dates in these regions advanced synchronously in El Niño years as weather conditions in the time period relevant for ice formation and break-up were strongly influenced by ENSO anomalies there. By contrast, lakes located further north were less affected by El Niño events[51]. Interannual changes in among-site coherence of ice events for lakes in North America, Finland, Russia, and Japan were smallest for ice-off dates, increased for ice-on dates, and were largest for ice duration[110]. The process of ice formation, primarily heat loss by the water mass of each site, depends on local conditions such as bathymetry distribution and water motion. On the contrary, thawing processes (primarily the energy balance of ice) are less site-dependent, leading to higher coherence of ice-off dates on a hemispheric scale[110]. Based on data from 184 study lakes located within the Northern Hemisphere, it was found that lakes with short ice duration have more variable freezing and thawing dates, and ice conditions generally were more variable in 1971–1990 as compared to 1951–1970[111].

Despite convincing evidence for synchronisation of processes among a variety of lakes exposed to the NAO or the ENSO (Fig. 1), the degree of such large-scale coherence is modulated by site-specific natural and anthropogenic factors (Table 1). Physical features such as lake morphology, depth, thermal structure, clarity, and ice conditions, are among the most relevant factors confounding spatial covariation among lakes[36,100]. Specifically, it was found that the NAO influence on water temperature profiles varied substantially among lakes in northeastern Germany according to differences in their heat-storage capacity[32] (Fig. 2, lower panel). Moreover, the phytoplankton spring bloom in Lake Constance usually establishes independently of the NAO (other than in Müggelsee and Lake Erken), because algal development is coupled to periods of low wind speed there[104,112]. In addition, the suggested NAO-driven synchronisation of the clear-water phase in Lake Constance and Müggelsee[104] is controversial. Analysis of a longer time series revealed that a warming trend in spring, not the NAO, was responsible for the earlier clear-water phase in Müggelsee[72]. This example illustrates well that climate-driven changes in ecosystem dynamics depend not only on the degree of a warming, but also on its detailed seasonal patterning[113].

**TABLE 1**
**Known Factors Potentially Preventing Lake Features and Lake-Internal Processes from NAO- or ENSO-Induced Synchronisation***

| Desynchronizing Factors | Affected Features | Lakes [References] |
|---|---|---|
| Geographic position, altitude | • Ice duration and break-up date<br>• Lack of ice cover; primary productivity | • Alpine lakes [37,51]<br>• Lake Tahoe [68] |
| Changes in the catchment | • Chemical processes | • English Lake District [31]; Wisconsin lakes [99,115] |
| Lake size and depth | • Water temperature<br>• Timing of clear-water phase<br>• Timing of lake mixis and phytoplankton spring bloom | • German lakes [32,72]<br>• Müggelsee [72]<br>• Lake Constance [71,104] |

* Example sites include those considered in this review.

Another example illustrating regional differences in lake responsiveness to large-scale climate variation are Castle Lake and Lake Tahoe. In the former, extremely high or low values of annual primary productivity occurred in ENSO years while productivity in Lake Tahoe was unaffected by the ENSO[68,114]. The main reason for that asynchrony was the absence of an ice cover at Lake Tahoe (see above, "Effects on Phytoplankton and Primary Production"). Interestingly, both the NAO[58,72] and the ENSO[68] affected primary productivity in either European or North American lakes as mediated by ice conditions, yet the detailed underlying mechanisms were different (snow cover probably played a more important role at Castle Lake).

The altitude at which lakes are situated may also have a distorting effect. For instance, longer periods of ice cover in high-altitude lakes dampened NAO-driven coherence in winter water temperatures among Austrian lakes; actually, coherence was greatest in low-lying lakes with infrequent and short ice cover periods[37].

One should note that spatial coherence may also vary through time. For instance, the level of chemical coherence among Wisconsin lakes increased when the observation period was extended by years with droughts, also suggesting that climate change may alter the structure of interlake synchronies[99,115]. In the English Lake District, nitrate concentrations were much more coherent among lakes in a decade with low anthropogenic input compared to a period when more fertiliser was used on local farms[31]. These examples illustrate that the actual length of a time series under examination may decide whether existing synchronies will be detected or will remain concealed.

Long-term synchronies among lakes may also be constrained by variations in the relationship between regional climate and the NAO or the ENSO, respectively (see Box 3). Moreover, transregional coherence is likely to vary seasonally, according to seasonal differences in the strength of NAO and ENSO impacts. NAO effects on European freshwaters, for example, usually disappear some time in spring, because traceable NAO influences on regional weather are restricted to the period from December to March or April[116]. Stratified lakes with high heat-storage capacity, however, are predisposed to store the NAO signal for most of the year in the hypolimnion[32,39,40] (Fig. 2, lower panel).

Furthermore, it may make a difference whether one applies the traditional winter NAO index or monthly indices. Actually, it was found that the strong impact of the winter NAO on year-around hypolimnetic water temperature in Stechlinsee (Germany) was not seen when using monthly NAO indices, except in case of the February index[117]. As a consequence, relevant

**BOX 3 — INTERACTIONS AMONG THE NAO, THE ENSO, AND CLIMATE CHANGE.** The positive trend of the NAO during the recent decades coincided with the concurrent winter warming in West and North Europe[116,150]. As the warming is expected to continue in the next century[177], it is tempting to extrapolate observed NAO impacts on ecosystems into the future. However, one has to consider that the role of the NAO is a rather controversial issue in the climate change debate[178,179], especially since its origin is not yet fully understood[145]. The possible mechanisms driving the NAO are currently the subject of considerable scientific interest, and it is still in discussion whether anomalies in the Atlantic sea surface temperatures[180] or variations in the strength of the polar stratospheric vortex[181] are its prime drivers. It is currently suggested that the NAO may be locked in an intense phase in the future, which may change its influence on regional climate in a manner unknown today. The Icelandic low and the Azores high may also move northwards so that the traditional NAO index (based on stations in Iceland and Portugal) will no longer match the decisive pressure constellation[182].

Furthermore, there is evidence that the NAO has not always shown the same behaviour in historical times. The amplitude of its variation might have increased since the end of the "Little Ice Age" around 1850[147,183], and its influence on air temperature exhibited pronounced interdecadal variability even during the 20$^{th}$ Century[103,184].

Similarly, both the activity and the periodicity of the ENSO have varied considerably during the past century. In particular, ENSO variability was reduced in the 1920–1960 epoch, and ENSO events were unusually frequent, intense, or persistent since 1976. The extreme event of 1997 and 1998 was probably outside the range of variability of the past centuries. It remains unclear whether this warm state will continue into the future, and, if so, its implications for regional climate change are still rather uncertain[145]. Furthermore, there is ambiguity about the question whether El Niño events are influenced by global warming, especially since they affect global temperature itself[185].

Overall, the NAO and the ENSO are key determinants of regional climate change around the Northern Hemisphere, although many questions remain about their future behaviour. Rather abrupt shifts in NAO and ENSO states — as have occurred recently — even may have greater consequences than a gradual increase in global mean temperatures, because ecosystems are particularly vulnerable to rapid climate change. Observed relationships between ecological variables and the NAO and ENSO are thus highly relevant contributions to the understanding of long-term ecosystem dynamics, yet they should always be interpreted in view of the historical and possible future variation of these ocean–atmosphere oscillations.

NAO–ecosystem relationships, and large-scale synchronies, might remain obscured if an inappropriate index is used. An example is provided for Lake Erken, where the timing of the phytoplankton spring peak was related significantly to the March NAO index but not to the winter index[58]. Similar differences may emerge when using different ENSO indices, but such effects are, to our knowledge, not yet documented for lakes.

To avoid such interpretation problems, one should focus on the comparative use of diverse climate indices. For example, it is recommended to filter air pressure records that are out of the

NAO phase; a corrected index based on these filtered pressure series will better represent the actual NAO state[118]. Additionally, it may be useful to employ further zonal and meridional climate indices, which cover more of the variability in regional climate than the rather broad NAO or ENSO indices[119,120].

Overall, the extent to which processes in lakes exhibit synchrony is still poorly understood and merits further investigation. For example, it would be challenging to study whether synchronicities in physical, chemical, and biological variables even exceed the immediate sphere of influence of the NAO. Such planetary wave-scale synchronies are likely, since the NAO appears as a regional manifestation of the Arctic Oscillation[37,121]. Changes in mid-latitude lakes may therefore occur in parallel with similar ones in Arctic lakes[61]. Furthermore, the NAO demonstrates teleconnections with the ENSO, thus the interplay between both oscillations may produce combined effects on ecosystems[122,123]. Hence, there is a need for comparative studies across latitudinal and longitudinal gradients in order to regionalize our understanding of climate–lake relationships and their spatio-temporal variability.

## PERSPECTIVES

The studies reviewed here provide ample evidence that the ongoing process of climate warming contributes substantially to changes in thermal properties of Northern Hemispheric freshwater ecosystems. These changes in turn are likely to influence the seasonal phytoplankton and zooplankton succession.

In order to assess the probable nature of changes in a warmer future climate, model-based predictions should be further encouraged[124]. Previous projections demonstrated that thermal conditions of lakes in temperate and subpolar zones are widely consistent with the observed interdecadal trends. For example, simulations indicated that epilimnetic temperatures of lakes would generally increase, while hypolimnetic temperatures would remain unchanged or decrease due to early stratification[125,126]. Higher water temperatures and prolonged periods of thermal stratification under climate warming scenarios were also predicted in a number of studies[38,127,128,129,130,131,132]. Furthermore, shorter periods of ice cover under climate warming scenarios were simulated for a variety of lakes[131,133], and these simulations were consistent with the observed trends described above. More effort is now required to model possible biotic responses to climate warming. Previous simulation studies have already indicated that fish([25,134]), phytoplankton, and zooplankton communities[135] are likely to exhibit shifts in their spatial or temporal distribution in response to climate warming.

In addition to deterministic simulation modeling, relatively simple approaches — e.g., (multilinear) regression models based on relationships derived from long-term impact studies — may be adequate to assess how key events (water temperatures, phenology of prominent events in the seasonal plankton succession, predator–prey relationships) will change, either synchronously or asynchronously, across geographical gradients and lake types. For example, future advancements in the timing of the phytoplankton spring bloom may be estimated based on robust statistical relationships with winter air temperatures (as predicted by regionalized General Circulation Models[136]) and the timing of ice break-up [53,56]. Potential differences among lake types should be captured effectively by specifying the most relevant desynchronising physical properties — such as lake size and depth, latitude and altitude, human population density of the drainage basin — as boundary conditions of such a statistical model. The models may be used, for example, to test the hypothesis that climate warming would tend to shift physical and biological patterns in northern dimictic lakes toward those that are currently characteristic for southern monomictic lakes[137]. Ideally, NAO and ENSO effects should be considered in model studies, although the role of these oscillations in the future is still rather speculative (see Box 3).

To fulfill the model demands, and to allow detection of critical changes in their initial stages[8], systematic long-term monitoring of lakes should be further promoted[17]. Sampling strategies should focus on variables that are sensitive to climate change, even if their vulnerability is documented for a few sites or is known from laboratory experiments only (e.g., primary productivity and secondary productivity of single species[81]). We feel furthermore that there is an urgent need for lake monitoring in less well-observed areas such as subpolar and subtropical regions (see Fig. 1). Based on such long-term observations, comparative studies and meta-analyses will be feasible[138].

Moreover, a cross-disciplinary perspective is required. Climate-driven synchronies even exist among limnetic and marine or terrestrial environments[139], and the interplay among climate warming, the NAO, and ENSO events produces a great many direct, indirect, and integrated responses across ecosystem types[140,141]. For example, winter warming and the NAO influenced simultaneously the spring diatom bloom in both European lakes (see above) and the English Channel[142], yet wind speed rather than ice conditions determined the extent of turbulent mixing in the latter. Furthermore, the NAO-driven advancement in tree phenology[95,96], and in the breeding time of reptiles and amphibians[143] are equivalent to the above-mentioned temporal shifts in the seasonal plankton succession in lakes.

Overall, spatio-temporal modeling approaches with varying degrees of complexity will enable us to deduce the primary ways that climate influences the dynamics in freshwater ecosystems around the Earth. This knowledge is an ultimate prerequisite for lake management and adaptation strategies, which aim to mitigate detrimental climate effects in a changing world.

## ACKNOWLEDGEMENTS

This study was funded by the Deutsche Forschungsgemeinschaft and the Leibniz-Institut für Gewässerökologie und Binnenfischerei. We are grateful to Norbert Walz and Christian Steinberg. The manuscript benefited from critical reading by Karl E. Havens and two anonymous reviewers.

## BIOSKETCHES

**Dieter Gerten,** Ph.D., is a Post-Doctoral Fellow at the Potsdam Institute for Climate Impact Research. His research interests include long-term development of limnic variables; global water balance and carbon cycling; climate–ecosystem interactions; hydrological modelling; and statistics and time series analysis.

**Rita Adrian**, Ph.D., is a Senior Scientist at the Leibniz Institute of Freshwater Ecology and Inland Fisheries. Her research interests include plankton ecology, long-term research, and climate–ecosystem relationships.
